# Neutrophil Depletion Changes the N-Glycosylation Pattern of IgG in Experimental Murine Sepsis

**DOI:** 10.3390/ijms25126478

**Published:** 2024-06-12

**Authors:** Kursat O. Yaykasli, Karin A. van Schie, René E. M. Toes, Manfred Wuhrer, Carolien A. M. Koeleman, Galyna Bila, Nazar Negrych, Georg Schett, Jasmin Knopf, Martin Herrmann, Rostyslav Bilyy

**Affiliations:** 1 Department of Internal Medicine 3, Rheumatology and Immunology, Friedrich-Alexander-Universität Erlangen-Nürnberg (FAU), Universitätsklinikum Erlangen, 91054 Erlangen, Germany; kursatyay@yahoo.com (K.O.Y.);; 2 Deutsches Zentrum für Immuntherapie (DZI), Friedrich-Alexander-Universität Erlangen-Nürnberg (FAU), Universitätsklinikum Erlangen, 91054 Erlangen, Germany; 3 Department of Rheumatology, Leiden University Medical Center, 2333 ZA Leiden, The Netherlands; 4 Center for Proteomics and Metabolomics, Leiden University Medical Center, 2333 ZA Leiden, The Netherlands; 5 Danylo Halytsky Lviv National Medical University, 79010 Lviv, Ukrainer.bilyy@gmail.com (R.B.); 6 Institute of Cellular Biology and Pathology ‘Nicolae Simionescu’, 050568 Bucharest, Romania; 7 Department of Pediatric Surgery, University Medical Center Mannheim, Heidelberg University, 68167 Mannheim, Germany

**Keywords:** neutrophils, neutrophil extracellular traps (NETs), IgG Fc glycosylation, sepsis

## Abstract

Sepsis is a life-threatening condition with a rising disease burden worldwide. It is a multifactorial disease and is defined as a dysregulated host response to infection. Neutrophils have been shown to be involved in the pathogenesis of sepsis by exacerbating inflammation. However, the exact effector mechanism of action still remains a mystery. Changes in the glycosylation pattern of the immunoglobulin G (IgG) Fc region are described for several diseases including meningococcal sepsis. In this study, we investigated the possible contribution of neutrophils and neutrophil implication, potentially related to degranulation or neutrophil extracellular trap (NET) formation in changing the IgG Fc N-glycosylation pattern in a murine sepsis model. We have measured the serum level of cytokines/chemokines and immunoglobulins, the serum activity of neutrophil elastase (NE), and analyzed the IgG Fc glycosylation pattern by Liquid Chromatography-Electrospray Ionization-Mass Spectrometry (LC-ESI-MS) and Lectin enzyme-linked immunosorbent assay (ELISA). We observed an increased activity of NE- and neutrophil-associated cytokines such as keratinocyte chemoattractant (KC) with the development of sepsis. Regarding the IgG Fc N-glycosylation, we observed an increase in fucosylation and α1,3-galactosylation and a decrease for sialyation. Interestingly, these changes were not uniform for all IgG subclasses. After depletion of neutrophils, we saw a change in the exposure of fucose and α2,6-linked sialic acid during the time course of our experimental sepsis model. In conclusion, neutrophils can influence changes in the IgG glycosylation pattern in experimental sepsis.

## 1. Introduction

Sepsis, often the cause of multiple organ failure, is a major medical emergency that can have life-threatening outcomes and is on the rise globally. The exaggerated and erratic immunological response of hosts against infections is the most recent definition for it [1,2]. However, its pathogenesis has not been clarified in detail. The involvement of a wide variety of processes including secretion of cytokines, activation of the coagulation cascade, and the involvement of neutrophil extracellular traps (NETs) have been described [3,4].

Neutrophils are the first line of defense against invading pathogens and have a high prevalence in the circulatory system. Therefore, they have been considered as a usual suspect in the etiology of sepsis, and the linkage between sepsis and neutrophils was established in 1995 [5]. Neutrophils can die through a wide range of mechanisms such as apoptosis, necrosis, pyroptosis, necroptosis, and the formation of NETs to perform their functions. Understanding the different types of neutrophil cell death is of pivotal importance in sepsis to develop new treatment strategies [6]. NETs have been shown to have detrimental effects on the etiopathogenesis of several diseases, especially by exacerbating inflammation [7,8], and being the primary death cause due to lung failure upon severe acute respiratory syndrome coronavirus type 2 (SARS-CoV-2) infection [9]. Excessive formation of NETs has also been demonstrated to cause deleterious effects on host tissue during sepsis [10,11]. The cytotoxic nature of NET components can exacerbate inflammatory responses by serving as damage-associated molecular patterns (DAMPs) when not adequately cleared, especially in endothelial cells. The activation/damage of endothelial cells has a central position in the understanding of sepsis development [12]. NETs have been demonstrated to cause endothelial dysfunction by activating matrix metalloproteinase-2 in addition to their direct impact on endothelial cell death [13,14]. Although there are several studies related to the role of neutrophils/NETs on endothelial cell dysfunction in sepsis as reviewed by Zhang et al. [15], the contribution of neutrophils/NETs to other cellular processes in sepsis is still unknown.

Immunoglobulins are Y-shaped glycoproteins. They belong to the adaptive immune system and perform a wide range of effector functions that are essential for humoral immunity [16]. The most common type in the circulatory system, immunoglobulin G (IgG), is highly involved in the adaptive immune response. There are four subclasses of IgG in humans (IgG1, 2, 3, and 4) and three in mice (IgG1, IgG1i, lgG2a/b/c, and IgG3) with different affinity for the fragment crystallizable (Fc) γ receptors and effector functions [17,18]. Altered serum levels of IgG are indicative not only of an ongoing infection but can also be caused by other circumstances including septic shock [19,20]. The level of IgG has also been linked to mortality in patients with severe sepsis [21,22]. In addition, the binding affinity of IgG to antigen through fragment antigen binding (Fab) and receptors through the Fc region is central for understanding their roles in disorders [23]. Human IgG contains a conserved glycan at Asn297 of the heavy chain, which influences the quaternary structure of the Fc and modulates binding to the Fc γ receptors [24,25]. It is well-established that the glycosylation pattern of the Fc region takes a vital role in inflammation and immunity. Neutrophils were shown to be able to change the composition of circulating immune complexes and alter exposed glycan residues [26]. A change in the IgG Fc glycosylation pattern in pediatric patients with meningococcal sepsis and in mice infected with different bacterial strains has also been demonstrated recently [27,28]. However, a thorough explanation of the reasons for this change has not yet been provided and the Fc glycosylation patterns have never been compared to the same individual before onset of sepsis.

This study investigates the impact of neutrophils on IgG N-glycosylation in a murine sepsis model, aiming to clarify the poorly understood mechanisms underlying sepsis pathogenesis. The advantage of our murine sepsis model is the possibility of directly comparing the N-glycosylation to basic levels before induction of sepsis. While clinical and preclinical studies have demonstrated a relationship between IgG N-glycosylation and sepsis [27,29], our research is the first to show the involvement of neutrophils in this process. Due to its rapid and potentially fatal progression, sepsis necessitates a comprehensive understanding of pre- and post-induction changes, which our model enables. Additionally, we pioneer the exploration of the relationship between IgG N-glycosylation and neutrophils in sepsis, highlighting the glycosylation pattern’s potential as a diagnostic and prognostic biomarker.

## 2. Results

### 2.1. Changes in IgG Glycan Exposure during the Time Course of Experimental Murine Sepsis

First, changes of IgG glycans before and after sepsis induction over time were analyzed by Lectin ELISA in a murine sepsis model in C57/BL6N mice before and during the course of sepsis induction. For this, IgG was captured from serum and exposed glycans were detected with the following lectins: (I) Aleuria aurantia lectin (AAL) for α1,6-linked fucose, (II) Lens culinaris agglutinin (LCA) for α-linked mannose, (III) Sambucus nigra agglutinin (SNA) for α2,6-linked sialic acid-galactose, and (IV) Polyporus squamosus lectin (PSqL) for α2,6-linked sialic acid on branched N-glycans [26,30]. 

**Figure 1:**
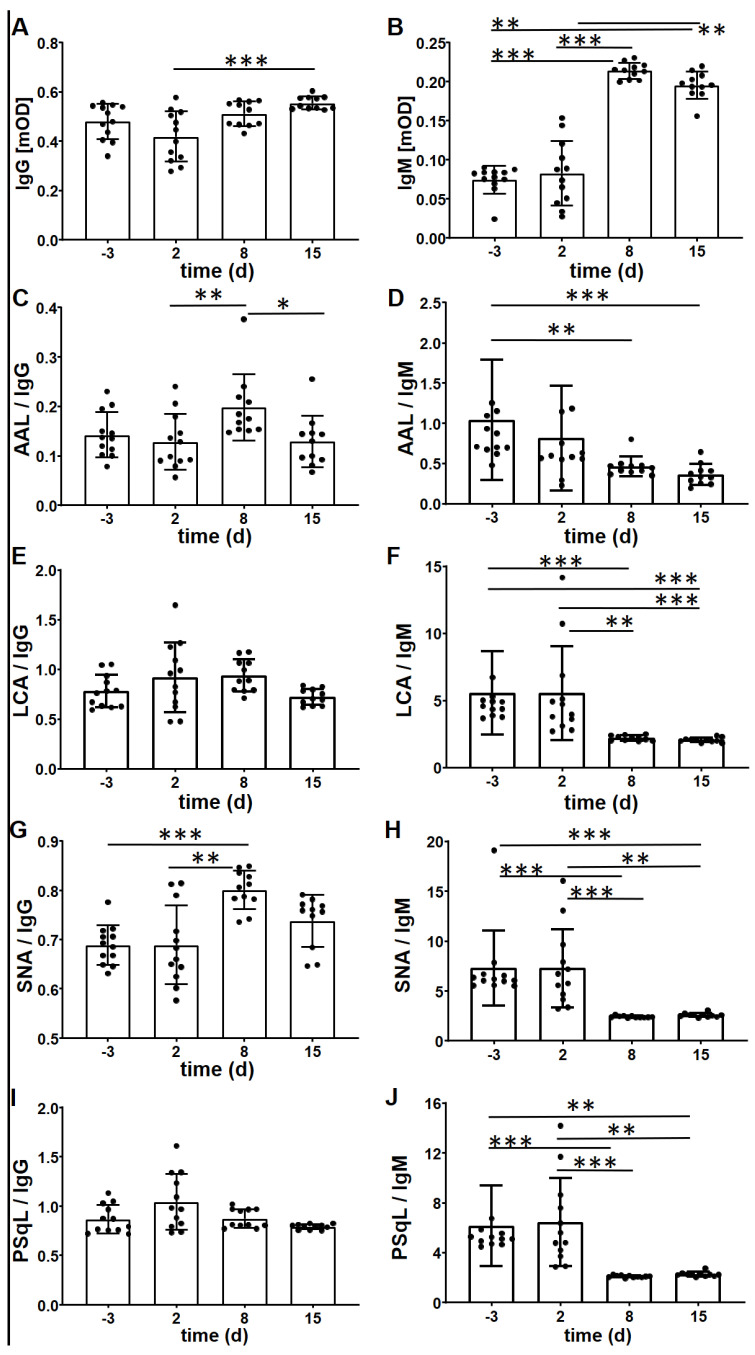
Immunoglobulin-associated glycans change within the course of experimental murine sepsis in C57/BL6N mice. Time course of the levels of IgG (**A**) and IgM (**B**) before and after sepsis induction is displayed. The serum immunoglobulin-associated glycans were detected by the lectins AAL (core α1,6-fucose) (**C**,**D**), LCA (fucosylated trimannose) (**E**,**F**), SNA (terminal a2,6-sialic acid) (**G**,**H**), and PSqL (terminal a 2,6-sialic acid of N-glycans) (**I**,**J**). The levels of lectins were normalized to the levels of IgG and IgM. Note that the glycosylation of both IgG and IgM changed substantially in the course of sepsis. AAL = Aleuria aurantia lectin; LCA = Lens culinaris agglutinin; SNA = Sambucus nigra agglutinin; pSqL = Polyporus squamosus lectin. Kruskall-Wallis test with Dunn’s multiple comparisons post-test was used to compare differences among time points (n = 12). * *p* < 0.05, ** *p* < 0.01, *** *p* < 0.001, •—represents individual data points.

Additionally, we determined the serum levels of IgG and IgM bound to IgG and normalized the lectin binding levels to these. Since IgM possesses abundant N-glycans and its levels during sepsis are usually increased, being a marker of systemic inflammation, the lectin signal was normalized to the IgG or IgM concentration in the sera (Figure 1, Table 1). The serum levels of IgG were slightly reduced two days after sepsis induction but then increased significantly until day 15 (Figure 1A). Exposure of α1,6-linked fucose on IgG as detected by AAL and α2,6-linked sialic acid as detected by SNA was highest on day eight after sepsis induction (Figure 1C,G). Interestingly, the binding of pSqL, also binding to α2,6-linked sialic acid on IgG, was fairly similar throughout the time course of sepsis (Figure 1I). The levels of exposed trimannose on IgG as detected by LCA stayed similar throughout the whole-time course (Figure 1E). Contrary, the levels of IgM were similar before and two days after sepsis induction before significantly increasing on day eight and 15 (Figure 1B). This results in a significant decrease of AAL, LCA, SNA, and PSqL binding when normalized to IgM quantities (Figure 1, Table 1). Since the IgG Fc glycosylation reportedly varies between mouse strains, we also analyzed the glycan exposure after sepsis induction in Balb/c mice. We observed a similar tendency as in the C57/BL6N mice (Figure S1, Table S1). When the glycan exposure was normalized to IgG, almost no significant changes were observed. However, significantly decreased binding of various lectins was evident especially on the 8th day of sepsis compared to before sepsis induction when the data was normalized to IgM (Figure S1D,F,H,J).

### 2.2. Changes in the IgG Glycopeptide Composition over the Time Course of Experimental Murine Sepsis

**Figure 2:**
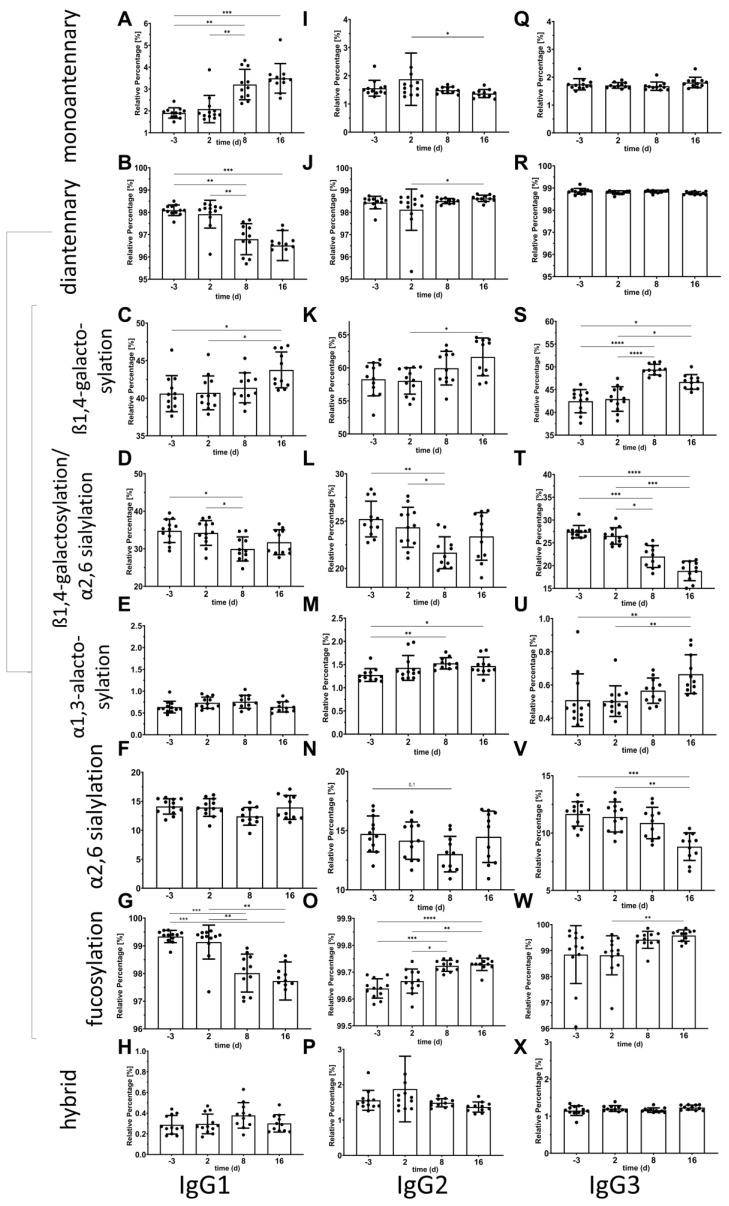
Changes in the composition of the IgG Fc glycans over the time course in experimental sepsis in the serum of C57/BL6N mice. Glycopeptide composition of the Fc region of IgG1 (**A**–**H**), IgG2 (**I**–**P**), and IgG3 (**Q**–**X**) was analyzed by Liquid Chromatography-Electrospray Ionization-Mass Spectrometry (LC-ESI-MS). The relative percentages of complex monoantennary and diantennary glycans of IgG Fc and the glycosylation traits: (I) ß1,4-galactosylation, (II) ratio of ß1,4-galactosylation and α2,6 sialylation, (III) α1,3-galactosylation, (IV) α2,6 sialylation, (V) fucosylation, and (VI) hybrid for diantennary glycans were calculated. Kruskall-Wallis test with Dunn’s multiple comparisons post-test was used to compare differences among time points (n = 12). * *p* < 0.05, ** *p* < 0.01, *** *p* < 0.001, **** *p* < 0.0001, •—represents individual data points.

The Fc-glycan composition of IgG purified from the sera of C57/BL6N mice during the time course of experimental sepsis was analyzed by mass spectrometry (LC-ESI-MS). The percentages of hybrid, complex mono-(A1), and complex di-(A2) antennary glycans as well as the different glycosylation traits of the A2 glycans were calculated separately for IgG1, IgG2 and IgG3 (Figure 2). 

There were no significant differences for the percentage of hybrid glycans for IgG1, IgG2 and IgG3 during the time course of experimental sepsis. The mono- and diantennary complex glycans changed significantly over time for IgG1 (Figure 2A–H), IgG2 (Figure 2I–P), and IgG3 (Figure 2Q–X). Regarding the glycosylation traits, the percentage of fucosylation significantly increased for IgG2 on days two and 15 after sepsis induction (Figure 2O) but neither for IgG1 nor for IgG3. The ß1,4-galactosylation levels also significantly increased for IgG1, IgG2 and IgG3, especially on day 15 after sepsis induction (Figure 2C,K,S). A similar trend was also observed for α1,3-galactosylation of IgG2 and IgG3 (Figure 2E,M,U) but not for IgG1. Interestingly, the sialylation of IgG3 is significantly decreased during the course of sepsis (Figure 2V), whereas the levels insignificantly decreased for IgG2 at day 8 (Figure 2N). This was also the case for the ratio of sialylation/galactosylation for IgG1, IgG2 and IgG3 (Figure 2D,L,T). For IgG1 the ratio of sialylation/galactosylation was significantly decreased on day eight after sepsis induction compared to the levels before, but the changes in sialylation were not significant (*p* = 0.13) and the levels of galactosylation increased over time (Figure 2C). Interestingly, during the time course of sepsis in Balb/c mice, no significant changes in IgG Fc N-glycosylation was observed.

### 2.3. Changes in IgG Subclasses and Cytokine Levels during the Time Course of Experimental Sepsis in C57/BL6N Mice

**Figure 3:**
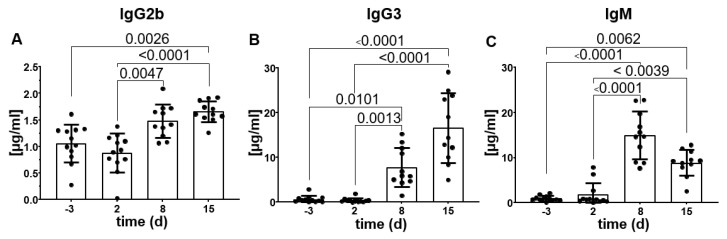
Changes in the serum immunoglobulin levels over the time course of experimental sepsis in C57/BL6N mice. Serum immunoglobulins concentrations were measured by LEGENDplex™ bead assay before and after sepsis induction in C57/BL6N mice (only significant data are shown, namely for IgG2b (**A**), IgG3 (**B**) and IgM (**C**)). Kruskall-Wallis test with Dunn’s multiple post-test was used to compare differences among time points (n = 12), •—represents individual data points.

Next, we wanted to analyze if changes in the IgG Fc-glycan exposure (Figure 1) and composition (Figure 2) were due to changes in the overall levels of the IgG subclasses in the serum during experimental sepsis in C57/BL6N mice. Isotyping of the murine immunoglobulins in the serum of the C57/BL6N mice before and after induction of sepsis showed a significant increase in the serum concentration of IgG2b, IgG3 and IgM (Figure 3). Whereas the increase of IgG2b (Figure 3A) was only moderate, the serum concentration of IgG3 was about 20-fold higher at day 15 after sepsis induction compared to the levels before (Figure 3B). A slight increase in total IgG was also seen in the lectin ELISA (Figure 1). The serum concentration of IgM was drastically increased on day eight after sepsis induction but already declined on day 15 (Figure 3C). Regarding Balb/c mice, similar but slightly different results were obtained (Figure S2). In C57/BL6N mice, the serum concentration of IgG2b, IgG3, and IgM were significantly changed over time while the changes in IgG2ac, IgG2b, and IgM with similar patterns were observed in Balb/c mice.

**Figure 4:**
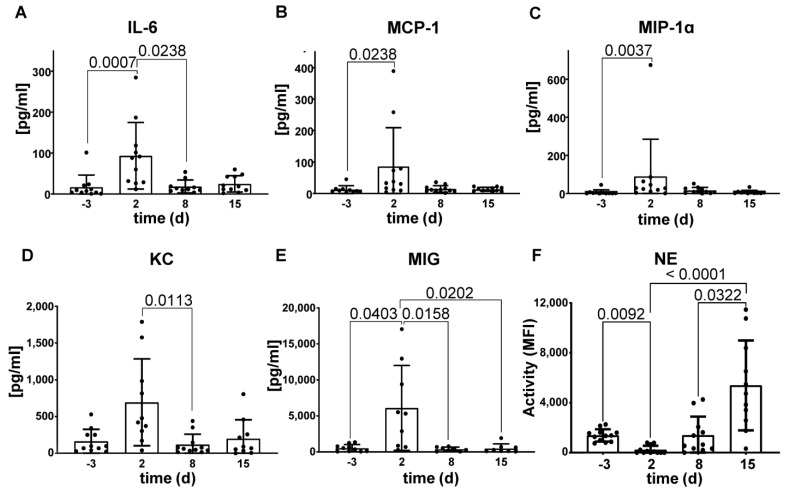
Changes in neutrophil elastase activity and serum levels of cytokines/chemokines in a model of experimental murine sepsis. (**A**–**E**) Sera levels of IFN-β, IFN-y, IL-10, IL-17A, IL-1β, IL-6, IP-10, KC, LIX, MCP-1, MIG, MIP-1a, and TNF-a were measured by a custom-made LEGENDplex™ cytokine/chemokine detection assay before and after sepsis induction in C57/BL6N mice. (**F**) Changes in the mean fluorescence intensity (MFI) levels of substrate converted by neutrophil elastase (NE) in the sera of C57/BL6N mice during the course of experimental sepsis over time in days. Kruskall-Wallis test with Dunn’s multiple comparisons post-test was used to compare differences among time points (n = 12). Only significant data of the cytokine/chemokine assay are shown, •—represents individual data points.

To better characterize the experimental sepsis model and get a more detailed insight into the immune response during the time course of the model, we analyzed the serum cytokine and chemokine composition. As depicted in Figure 4, the serum cytokines interleukin (IL)-6, Monocyte Chemoattractant Protein-1 (MCP-1) and Macrophage Inflammatory Protein-1 Alpha (MIP-1α), keratinocyte chemoattractant (KC) and Monokine induced by Gamma-Interferon (MIG) were significantly elevated two days after sepsis induction compared to the concentrations before. This increase in the concentrations was almost normalized again at day eight after sepsis induction. Other cytokines such as tumor necrosis factor alpha (TNF-α) or the chemoattractant protein Interferon gamma-induced protein 10 (IP-10) indicating inflammation were also increased, but changes did not reach significance (Figure S3). In contrast, no significant changes for cytokines were observed in Balb/c mice. Since the significant changes observed in the cytokine/chemokine levels in C57/BL6N mice are all in mediators involved in neutrophil recruitment and migration and neutrophils/NETs are known to play a pivotal role in sepsis, we wanted to further clarify their impact during the time course of experimental sepsis.

Therefore, the activity of neutrophil elastase (NE), a key enzyme of neutrophils and associated with NETs, was measured before and after sepsis induction in the sera of C57/BL6N mice (Figure 4F). The NE activity decreased significantly (*p* = 0.0092) at the 2nd day after sepsis induction and started to increase significantly after that (*p* < 0.0001 and *p* = 0.0044, respectively, 2nd day vs. day 15). Similar tendency was also observed in Balb/c mice (Figure S4).

### 2.4. Neutrophil Depletion Changed the Exposure of IgG Glycans in a Model of Murine Sepsis

**Figure 5:**
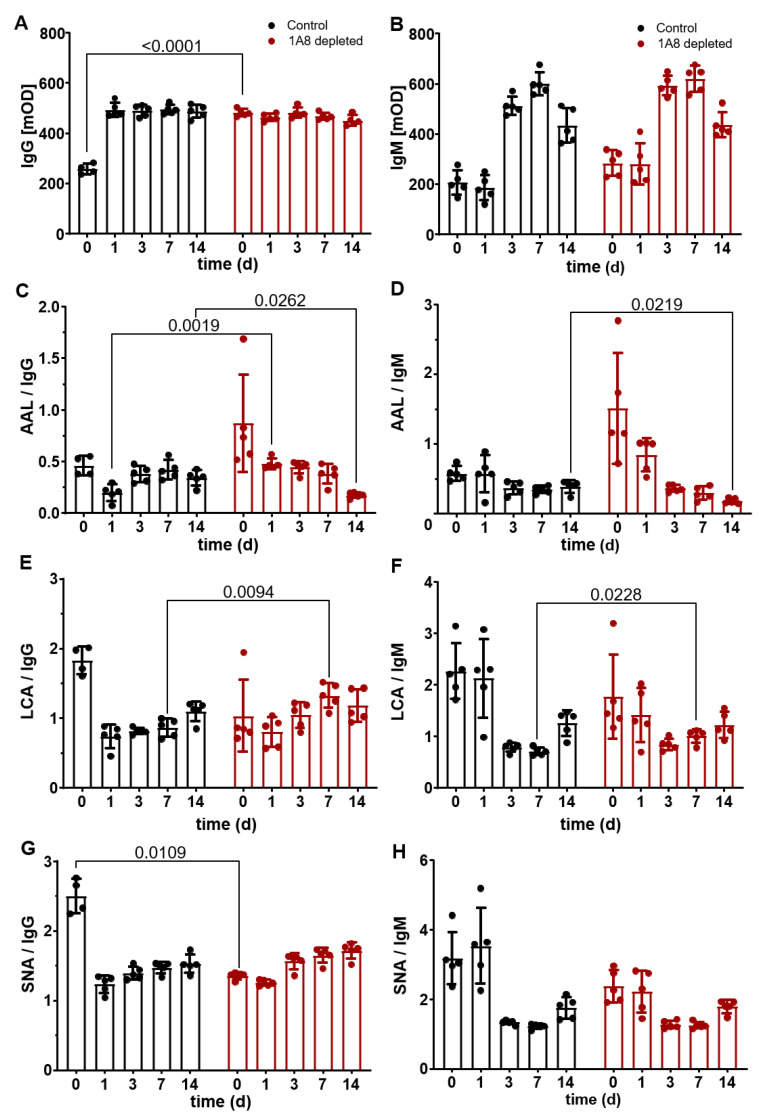
Depletion of neutrophils changed the glycosylation of IgG and IgM bound to IgG. Neutrophils were depleted using the 1A8 depletion antibody (n = 5) or an isotype control (n = 5) during the time course of experimental sepsis in C57/BL6N mice. Next to the serum levels of IgG and IgM bound to IgG, the exposure of the IgG glycans was assessed by lectin ELISA employing the following lectins: (I) AAL (α1,6-linked fucose), (II) LCA (α-linked mannose), and (III) SNA (α2,6-linked sialic acid). The levels were normalized to IgG (**A**,**C**,**E**,**G**) and IgM (**B**,**D**,**F**,**H**). Two-way ANOVA (or Mixed-effects analysis) with Sidak multiple comparisons post-test was used to compare differences among time points.

Since we saw a significant increase in neutrophil-related cytokines/chemokines and activity of NE in the sera of mice during the time course of experimental sepsis, we wanted to assess the effect of neutrophil depletion on the exposure of IgG glycans during the experimental sepsis in C57/BL6N mice. We therefore depleted neutrophils from the circulation of these mice using the 1A8 depletion antibody (and compared data to isotype control treated group) during the time course of sepsis. Then, the exposure of IgG glycans, namely (I) α1,6-linked fucose (by AAL), (II) α-linked mannose (by LCA), and (III) α2,6-linked sialic acid (SNA), was assessed by ELISA and normalized to either IgG or IgM bound IgG (Figure 5). Interestingly, the depletion of neutrophils resulted in a decrease in total IgG in isotype-treated mice on day 0 compared to 1A8-neutrophil-depleted mice (Figure 5A), whereas the levels of IgG bound IgM changed similarly over time (Figure 5B). 

The exposure of α1,6-linked fucose detected by AAL was significantly higher at the 1st day of sepsis in the serum of 1A8-neutrophil-depleted mice than in the control mice when normalized to IgG (*p* = 0.0019). In contrast, AAL binding was significantly lower at day 14 in the serum of 1A8-neutrophil-depleted mice than in the control when normalized to both IgG (*p* = 0.0262) and IgM (*p* = 0.0219) (Figure 5C,D). LCA, binding to (fucosylated) α-linked mannose, was also significantly increased on day seven in the serum of 1A8-neutrophil-depleted mice compared to control mice in both IgG (*p* = 0.0094) and IgM (*p* = 0.0228) normalized ELISAs (Figure 5E,F). The level of terminal α2,6-sialic acid-bound galactose detected by SNA was significantly lower before the induction of sepsis in the serum of 1A8-neutrophil-depleted mice (*p* = 0.0109) compared to controls (Figure 5G); no significant differences were observed for the binding of SNA normalized to IgM (Figure 5H).

## 3. Discussion

The excessive inflammatory response is the most prominent aspect of sepsis, which has a complicated etiopathogenesis. Since dysregulated responses to infection cause exaggerated inflammation, neutrophils become the usual suspect for driving this inflammation [31,32]. NETs as a functional structure formed by neutrophils to carry out their functions have also been linked to sepsis [33]. While clinical and preclinical data linked IgG N-glycosylation to the severity and outcomes of sepsis [27,28], our research is the first to directly examine the role of neutrophils in modulating this process. In addition, we are establishing a new line of research on the interplay between neutrophils and IgG glycosylation in sepsis, with the potential to reveal glycosylation patterns as valuable diagnostic and prognostic biomarkers.

Since the development of sepsis results from the simultaneous occurrence of heterogeneous pathologies, the issue of care standardization for patients is still under debate by clinicians [34]. To overcome this issue, the etiopathogenesis of sepsis needs to be well understood. The main drawback of studies using patient samples is the difficulty in pinpointing the precise timing of sepsis onset and what stage of sepsis the patients were in. Basically, the studies are conducted by merely comparing the patients with healthy donors [35]. Therefore, a model of experimental sepsis in mice was chosen to overcome this issue. Due to the experimental induction of sepsis, we were able to draw blood from the mice before and at certain time points after onset of disease. A recent study also found that human transcriptome responses to inflammatory conditions are strikingly similar to mouse models [36]. Two different mouse strains (C57/BL6N and BALB/c) were used concurrently in this study since the glycosylation patterns were reported to be slightly different [37].

While sepsis was originally attributed to a cytokine storm, research has shown that both pro- and anti-inflammatory agents are activated in sepsis [34]. However, IL-1β, TNF-α and IL-6 are considered to be key players in sepsis [38,39]. In fact, IL-6 plays a role in triggering inflammation in sepsis by mobilizing neutrophils [40]. Our model reflects this by showing peaks in markers such as IL-1β, IL-6, TNF-α, MCP1, MIP-1α, KC, and MIG. This is followed by declines over time (Figure 4). In particular, MCP-1 and MIP-1α as CC chemokines are hallmarks of sepsis and can serve as potential biomarkers [41,42].

In addition, these agents play a crucial role in recruitment and activation of neutrophils. MCP-1 and MIP-1α reportedly activate and prime neutrophils [43,44]. Furthermore, the involvement of the chemokines including KC and MIG in both sepsis and neutrophil activation has been demonstrated. KC attracts neutrophils while MIG is associated with diapedesis [39,45]. Cytokines and chemokines also interact with immunoglobulins, a crucial component of immunity during sepsis development. In our sepsis model, we observed that cytokine/chemokine production peaked followed by an increase in IgG and IgM levels [46]. Research suggests links between chemokines such as MIP 1α, glycosylation, and inflammatory cytokines, with the latter putatively affecting IgG N-glycosylation [47].

While immunoglobulin levels are considered potential biomarkers, studies on IgG in sepsis have shown inconsistent results [48]. This could be due to fluctuations in the measurement timing. Our results show an increase in IgG2b, IgG3 and IgM in the late stages of our model, indicating fluctuations in immunoglobulin levels during the progression of sepsis (Figure 3). This highlights the importance of the correct timing of drug administration in the treatment of sepsis [49].

Glycosylation is another modulating factor in determining the functionality of immunoglobulins, especially for IgG. The unique N-glycosylation pattern in the Fc region has been demonstrated to associate with inflammatory autoimmune conditions [50,51]. In addition, aging [52,53] and several pathological processes including coronavirus infection [54,55], inflammatory arthritis [56], type 1 diabetes [57], inflammatory bowel disease [58], and active tuberculosis infections [59] have been linked to changes in the N-glycosylation profile of the IgG Fc-region [60]. Finally, the altered Fc-glycosylation pattern in total plasma IgG was demonstrated to associate with pediatric meningococcal sepsis for the first time in 2018 [28]. In this study, the sialylation per galactose of IgG2/3 was found to be associated with the severity and outcome of sepsis. In addition, statistically significant differences in certain types of glycosylation (galactosylation, α1,3-galactosylation, complex A1 and A2) have been found in our model over time (Figure 2). In addition to analyzing the specific composition of the N-glycans, we also employed lectin ELISA to address the exposure of distinct sugar moieties such as fucose or sialic acid. This exposure of the different sugar moieties is important for binding features. The exposure of fucose was significantly increased on day eight after sepsis induction (Figure 1B), which was also true for the exposure of α2,6-linked sialic acid as determined by SNA but not by PSqL binding (Figure 1D,E). The change in exposure of α2,6-linked sialic acid is more evident in IgM-normalized data in a time-dependent manner (Figure 1D,E). Since the most significant changes occur on the 8th day of sepsis induction, it can be concluded that the changes of glycosylation mostly occurred in the middle stages of sepsis, or at least several days after the induction of sepsis. This data makes it compulsory to consider the stage of sepsis in glycosylation-targeted sepsis treatment.

Neutrophils are effector cells of the innate immune system. However, they also engage in cross-talk with components of the adaptive immune system in order to fight pathogens efficiently. These complex interactions may result in chronic inflammation in the case of prolonged presence or disturbed degradation of NETs [31,61]. The prothrombotic activity of NETs has been shown to contribute to the development of sepsis [32]. In addition, the levels of both NETs and their markers, such as neutrophil elastase, have been reported to increase excessively in sepsis and correlate with its severity [33,62,63]. Similarly, we found an increased NE activity in the late stage of sepsis in our murine model (Figure 4F). The fact that neutrophils/NETs play a critical role in the development/progression of sepsis brought the idea of neutrophil depletion to the agenda in the treatment of sepsis. However, it is important to identify more precisely the potential impacts of neutrophil depletion on crucial processes like glycosylation pattern in sepsis. Our follow-up research also revealed that neutrophil depletion had an effect on the IgG glycosylation profile in our experimental sepsis model. The neutrophil depletion changed especially the exposure of fucose (detected by specific binding of AAL) (Figure 5C,D), α-linked (fucosylated) mannose (detected by specific binding of LCA) (Figure 5E,F), and α2,6-linked sialic acid (detected by specific binding of SNA (Figure 5G). A higher level of fucose exposure was observed during sepsis stages when NE was also increased. This was in good accordance to the LC-ESI-MS data where sepsis was accompanied by an increase in fucosylated two-antenna-bearing (A2) glycans. At the same time, the amount of A1 glycans almost doubles during the course of sepsis; since they are sialylated, they originate from A2 (probably due to trimming of one glycan branch). In [26] it was demonstrated that incubation of human sera with NETs resulted in huge exposure of fucose residues associated with the immunoglobulin light chain when incubation was done at 37 °C, but not at 4°C. Protein paucimannosylation (appearance of trimmed oligomannose glycan) was reported as a significant host-derived molecular signature of neutrophil-rich sputum from pathogen-infected human lungs, and was negligible in pathogen-free sputum [64]. Thus, neutrophil-associated enzymatic release, observed during sepsis, can be a big contributor to the observed altered glycan phenotype.

The N-glycosylation level of the Fc region changes the binding features of IgG and affects whether IgG has a pro-inflammatory or anti-inflammatory effect. Therefore, the N-glycosylation pattern is directly associated with immunity/inflammation [65,66]. In addition, the changes in glycosylation patterns with a notable increase in fucose exposure correlate with the late stage of sepsis when neutrophil elastase activity is elevated. This made neutrophil-associated enzymatic release a potential target in studies of the etiopathogenesis of sepsis [65,67]. However, the relationship between this correlation has not been fully revealed. In addition, neutrophil depletion has potential for use as a therapeutic approach [68], but its effect on mechanisms like IgG Fc N-glycosylation in sepsis has not been fully understood [31,69]. In this study, the effects of neutrophil depletion on the N-glycosylation pattern have been revealed for the first time. Our investigation is the first to demonstrate the impact of neutrophil depletion on the IgG glycosylation profile in experimental sepsis. This finding not only underscores the intricate interplay between neutrophils and IgG glycosylation but also opens new avenues for exploring therapeutic targets in sepsis management. Understanding these glycan markers could lead to the development of personalized diagnostic and treatment strategies, harnessing the complex relationship between neutrophils, IgG glycosylation, and immune responses to improve sepsis outcomes. However, this warrants additional experiments to further strengthen the notion that neutrophils are involved in the regulation/modulation of IgG glycosylation patterns.

## 4. Materials and Methods

### 4.1. Murine Sepsis Model

Black C57/BL6N (n = 22) and white Balb/c mice (n = 6) were bred at the animal house of Danylo Halytsky Lviv National Medical University (Lviv, Ukraine). The animals were kept in a clean environment, with water and food available ad libitum. Studies involving animals, including housing and care, method of euthanasia, and experimental protocols were approved by the Ethical committee of Danylo Halytsky Lviv National Medical University, protocols 20191216/10, 20180226/2, 20170223/5 & 20130624/6, and all experiments were designed to comply with the principles of the 3Rs (Replacement, Reduction and Refinement). Polymicrobial sepsis was induced in adult mice using a modification of the cecal slurry method, as described by Sam et al. [70]. Briefly, an adult C57/BL6N female mouse aged 7 to 10 weeks was killed, and a midline laparotomy was performed to isolate the cecum. Using scissors, the cecum was opened at its most distal point, and the cecal contents were expressed and weighed. A suspension of the cecal contents and 5% dextrose was filtered and used for injection with a final concentration of 80 mg/mL. The cecal slurry was briefly vortexed before injection to create a homogenous suspension and was used within 2 h of preparation. Before the experiments involving blood collection for LC-ESI-MS, 12 mice were divided into three equal subgroups. Blood was collected from all groups before the experiment (day −3), at day 0 bacterial inoculation was performed, and then blood was collected from subgroups at days 1,2,3 (designated as 2), at days 7, 8, 9 (designated as 8), and days 14, 15, 16 (designated as 15).

### 4.2. Neutrophil Depletion in Murine Sepsis

Mice (n = 5 in each group) were injected intraperitoneally with neutrophil-depleting antibody anti-Ly6G (clone 1A8, rat IgG2a/k) or the same isotype κ Isotype Control [2A3, both BioXCell, Lebanon, NH, USA] antibody using 500 μg antibody per mouse every 2 or 3 days over the entirety of the indicated depletion periods.

### 4.3. Lectin ELISA for IgG and IgM Glycosylation Analyses

To analyze the exposure of IgG glycans, 96-well Nunc MaxiSorp™ 96-well ELISA plates (Thermo Fisher Scientific, Waltham, MA, USA) were coated with AffiniPure F(ab’)₂ Fragment Goat Anti-mouse IgG (H + L) (Jackson ImmunoResearch Europe, Ely, UK) overnight in coating buffer pH 9.6 at +4 °C. The next day, plates were washed three times with TBS-T and blocked with deglycosylated gelatin blocking buffer for 2 h at RT. After another washing step, serum was added at a dilution of 1:1000 in TBS-T for 1 h at RT. The plates were then washed three times before incubation with biotinylated lectins Aleuria aurantia Lectin (AAL) (specific for α1,6-linked fucose), Lens culinaris agglutinin (LCA) (specific for α-linked mannose), Sambucus nigra Lectin (SNA) (specific for α2,6-linked sialic acid, both Neu5Ac and Neu5Gc) (all Vector Laboratories, Burlingame, CA, USA), and Polyporus squamosus lectin (PSqL) (specific for α2,6-linked sialic acid to galactose in biantennary N-glycans) (kind gift of Prof. Gabius) for 1 h at RT. Binding of the specific lectins to IgG or IgM glycans was detected after another washing step by incubation with Streptavidin HRP (Thermo Fisher Scientific) for 1 h at RT. For the detection of bound IgG, goat anti-mouse IgG Fc HRP- (both Southern Biotech, Birmingham, AL, USA) were added for 1 h at RT. For the detection of IgG-bound IgM immune complexes, goat anti-mouse IgM HRP-conjugated antibody (both Southern Biotech, Birmingham, AL, USA) were added for 1 h at RT. For the detection of IgM levels, the F(ab’)2 Goat Anti-Mouse IgM) (Jackson ImmunoResearch Europe, Ely, UK) were sorbed on plate as described above and then goat anti-mouse IgM HRP-conjugated antibody (Jackson ImmunoResearch Europe, Ely, UK) was used for detection. After a final washing step, the colorimetric reaction was developed using the TMB substrate set (BioLegend, San Diego, CA, USA) and the reaction was stopped using 25% sulfuric acid (AppliChem, Darmstadt, Germany). The optical density (OD) was then read at 450 nm with 620 nm reference wavelengths using the SunriseTM absorbance microplate reader (Tecan, Männedorf, Switzerland). The given ODs were analyzed using Microsoft Excel Office 2019 (Microsoft Corporation, Redmond, WA, USA). To compensate for marginal differences in the signal of different ELISA plates, we always calculated the lectin/IgG ratio. Lectin/IgG ratios were visualized using GraphPad Prism 9 (GraphPad Software, San Diego, CA, USA).

### 4.4. Measurement of Neutropil Elastase Activity in Serum

25 µL of serum was added to 175 µL of PBS + 2.5 µL 15mM fluorogenic substrate MeOSuc-AAPV-AMC (N-Methoxysuccinyl-Ala-Ala-Pro-Val-7-amido-4-methylcoumarin, Santa Cruz Biotechnology, sc-201163) in black 96-well plates (ThermoFischer Scientific, 137101). Fluorescent readings at 37 °C were collected on a Perkin Elmer HTS7000Plus Bio Assay reader using the corresponding filter set (excitation 360/50 nm, emission 465/60 nm) for 4 h with a 15 min interval. Human leukocyte elastase (Abcam ab91099) and 4-methylcoumarin (Sigma-Aldrich, St. Louis, MO, USA) were used for calibration control in determination of specific activity. Assays were performed with technical duplicates.

### 4.5. LC-ESI-MS Analyses of IgG Fc Glycosylation

To characterize the glycosylation of IgG Fc, we additionally employed Liquid chromatography-electrospray ionization mass spectrometry (LC-ESI-MS). Total IgG was purified from serum using Protein G Sepharose (GE Healthcare, Chicago, IL, USA) and the IgG eluates were digested with sequencing grade modified trypsin (Promega) overnight at 37 °C. The IgG peptides were then analyzed for specific Fc-glycans as described in Knopf et al. [71]. Briefly, detected mass spectra were analyzed for the typical retention times of IgG1, IgG2 and IgG3 glycopeptides using the Compass DataAnalysis software V5.0 (Bruker, Billerica, MA, USA) and specific glycans were extracted from the data using LaCyTools [72]. The quality of mass spectra was first evaluated based on total signal intensities per glycopeptide cluster and analyte curation was performed using the signal to noise ratio (S/N ≥ 9) and isotopic pattern quality (mass error between −20 and 20 ppm; deviation from theoretical isotopic pattern ≤ 20%). The analysis of all samples was continued only with glycopeptides that were present in at least four samples of one timepoint. Glycosylation traits were calculated per subclass, based on the glycopeptide composition in Table 2 as described in de Haan et al. [37]. Glycosylation of IgG was calculated using Microsoft Excel Office 2019 and visualized using GraphPad Prism 9 (GraphPad Software). 

Glycan traits were calculated for A2 glycans, using the following formulas: 

Fucosylation: SUMIF(IgGx*F*)/SUMIF(IgGx*), where x is the subclass identifier, asterisks are wildcards, and F indicates fucosylation.

Galactosylation: (0.5 × SUMIF(IgGxH4*) + SUMIF(IgGxH5* + IgGxH6* + IgGxH7*))/SUMIF(IgGx*), where x is the subclass identifier, asterisks are wildcards, H4 indicates the presence of one galactose (next to the 3 mannoses), and H5, H6 and H7 indicate the presence of two or more galactoses (next to the 3 mannoses).

Bisection: SUMIF(IgGx*N5*)/SUMIF(IgGx*), where x is the subclass identifier, asterisks are wildcards, and N5 indicates the presence of a bisecting GlcNAc.

Sialylation: (0.5×SUMIF(IgGx*S1) + SUMIF(IgGx*S2))/SUMIF(IgGx*), where x is the subclass identifier, asterisks are wildcards, S1 indicates the presence of one sialic acid, and S2 indicates the presence of two sialic acids.

α1,3-Galactosylation: (0.5 × SUMIF(IgGx*H6*) + SUMIF(IgGx*H7*))/SUMIF(IgGx*), where x is the subclass identifier, asterisks are wildcards, H6 indicates the presence of one α1,3-galactose, and H7 indicates the presence of two α1,3-galactoses.

**Figure 6:**
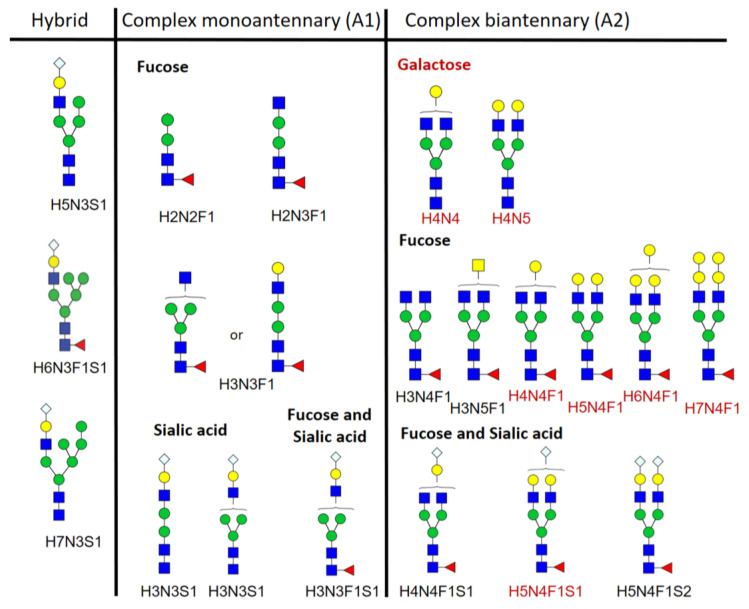
Schematic representation of all glycoforms we studied. The forms with terminal galactose are labeled in red. Please note that all forms bearing sialic acid also contain subterminal galactose residue. N-acetylglucosamine (blue rectangle), fucose (red triangle), mannose (green circle), galactose (yellow circle), sialic acid (light blue rhombus).

A schematic representation of the studied glycoforms can be found in Figure 6.

### 4.6. Measurement of Serum Immunoglobulin (Ig) Levels

Serum was analyzed for the concentration of IgA, IgM, IgG1, IgG2a/c, IgG2b, and IgG3 using the LEGENDplex™ Mouse Immunoglobulin Isotyping Panel (BioLegend) according to manufacturer’s instructions with minor modifications. Briefly, diluted serum samples, standard, beads and detection antibody were added together in assay buffer and incubated for 2 h at RT shaking. Then, streptavidin-PE was added and incubated for another 30 min at RT shaking. After washing twice with the provided wash buffer, the samples were read on a Gallios™ Flow Cytometer (Beckman Coulter, Brea, CA, USA) and analyzed using Kaluza Analysis Software V2.1 (Beckman Coulter). Individual immunoglobulin concentrations were then calculated using Microsoft Excel Office 2019 (Microsoft Corporation) and visualized using GraphPad Prism 9 (GraphPad Software).

### 4.7. Measurement of Serum Cytokine/Chemokine Levels

Serum cytokine and chemokine concentrations were analyzed using a custom-made 13-plex LEGENDplex™ kit (BioLegend) containing the following cytokines/chemokines: TNF-α, IFN-β, IFN-γ, IL-10, IL-17A, IL-1β, IL-6, IP-10, LIX, monocyte chemoattractant protein 1 (MCP1), macrophage inflammatory proteins (MIP-1α), keratinocyte chemoattractant (KC), and monokine induced by interferon-γ (MIG) according to manufacturer’s instructions. Briefly, serum samples, standard, beads and detection antibody were added together in assay buffer and incubated for 2 h at RT shaking. Then streptavidin-PE was added and incubated for another 30 min at RT shaking. After washing twice with the provided wash buffer, the samples were read on a Gallios™ Flow Cytometer (Beckman Coulter) and analyzed using Kaluza Analysis Software V2.1 (Beckman Coulter). Individual cytokine/chemokine concentrations were then calculated using Microsoft Excel Office 2019 (Microsoft Corporation) and visualized using GraphPad Prism 9.0.2 (GraphPad Software).

### 4.8. Statistical Analysis

Differences among certain time points during the time course of murine sepsis were evaluated with the Kruskall-Wallis test with multiple sample correction by Dunn or Two-way ANOVA (Mixed-effects analysis) with Sidak post-test for multiple comparisons. All analyses were performed using Prism 9.0.2 (GraphPad Software). *p* values lower than <0.05 were considered significant.

## Figures and Tables

**Table 1:** Significant changes in the exposure of immunoglobulin-associated glycans within the course of experimental murine sepsis in C57/BL6 mice. Significant values are displayed in bold. Kruskall-Wallis test with Dunn’s multiple comparisons post-test was used to compare differences among time points (n = 12).

Time	IgG	IgM	AAL/ IgG	AAL/ IgM	LCA/ IgG	LCA/ IgM	SNA/ IgG	SNA/ IgM	PSqL/ IgG	PSqL/ IgM
**day −3 vs. 2**	n.s.	n.s.	n.s.	n.s.	n.s.	n.s.	n.s.	n.s.	n.s.	n.s.
**day −3 vs. 8**	n.s.	**<0.000**	n.s.	**0.005**	n.s.	**0.000**	**0.000**	**<0.000**	n.s.	**<0.000**
**day −3 vs. 15**	n.s.	**0.002**	n.s.	**<0.000**	n.s.	**<0.000**	n.s.	0.001	n.s.	**0.003**
**day 2 vs. 8**	n.s.	**<0.000**	**0.010**	n.s.	n.s.	**0.002**	**0.001**	**<0.000**	n.s.	**<0.000**
**day 2 vs. 15**	**0.001**	**0.005**	n.s.	n.s.	n.s.	**<0.000**	n.s.	0.002	n.s.	**0.004**
**day 8 vs. 15**	n.s.	n.s.	**0.017**	n.s.	n.s.	n.s.	n.s.	n.s.	n.s.	n.s.

Significant values are displayed in bold. Kruskall-Wallis test with Dunn’s multiple comparisons post-test was used to compare differences among time points (n = 12).

**Table 2:** Glycopeptide composition and proposed structures used for calculation of the glycosylation traits. H—number of hexoses, N—number of N-acetylglucosamines (GlcNAc), F—number of fucoses, S—number of sialic acids.

IgG1	IgG2a/b/c	IgG3	Glycan Structure
-	-	H2N2F1	
H2N3F1	-	H2N3F1	
H3N3F1	H3N3F1	H3N3F1	G0F-N
H3N3S1	-	-	
H4N3F1	H4N3F1	H4N3F1	G1F-N
-	H4N3F1S1	-	G1FS-N
H4N3S1	-	-	
H5N3S1	-	-	
H6N3F1S1	-	-	
H3N4F1	H3N4F1	H3N4F1	G0F
H3N5F1	-	-	
-	H4N4	-	
H4N4F1	H4N4F1	H4N4F1	G1F
H4N4F1S1	H4N4F1S1	H4N4F1S1	G1FS
-	H5N4	-	
H5N4F1	H5N4F1	H5N4F1	G2F
H5N4F1S1	H5N4F1S1	H5N4F1S1	G2FS
H5N4F1S2	H5N4F1S2	H5N4F1S2	G2FS2
H6N4F1	H6N4F1	H6N4F1	G3F
H6N4F1S1	H6N4F1S1	H6N4F1S1	G3FS
-	-	H7N3S1	
-	H7N4F1	H7N4F1	G4F

To calculate the glycosylation traits, the glycopeptides were first separated into hybrid, complex monoantennary, and diantennary IgG (Table 3).

**Table 3:** Glycopeptide composition of different IgG forms. H—number of hexoses, N—number of N-acetylglucosamines (GlcNAc), F—number of fucoses, S—number of sialic acids.

Hybrid	Complex Monoantennary	Complex Diantennary
H5N3S1 H6N3F1S1	H2N2F1 H2N3F1	H3N4F1
H7N3S1	H3N3F1	H3N5F1
	H3N3S1	H4N4
	H4N3F1	H4N4F1
	H4N3S1	H4N4F1S1
	H4N3F1S1	H5N4
		H5N4F1
		H5N4F1S1
		H5N4F1S2
		H6N4F1
		H6N4F1S1
		H7N4F1

## Data Availability

Data are contained within the article or Supplementary Materials.

## Supplementary Materials

The following supporting information can be downloaded at: https://www.mdpi.com/article/10.3390/ijms25126478/s1.
