# Supplementary material for: Neutrophil Depletion Changes the N-Glycosylation Pattern of IgG in Experimental Murine Sepsis
Source: Int J Mol Sci. 2024 Jun 12;25(12):6478. doi: 10.3390/ijms25126478 (PMC11203722; doi:10.3390/ijms25126478)
Supplement: Supplementary file 1 [file ijms-25-06478-s001.zip › ijms-2993225-supplementary.pdf]

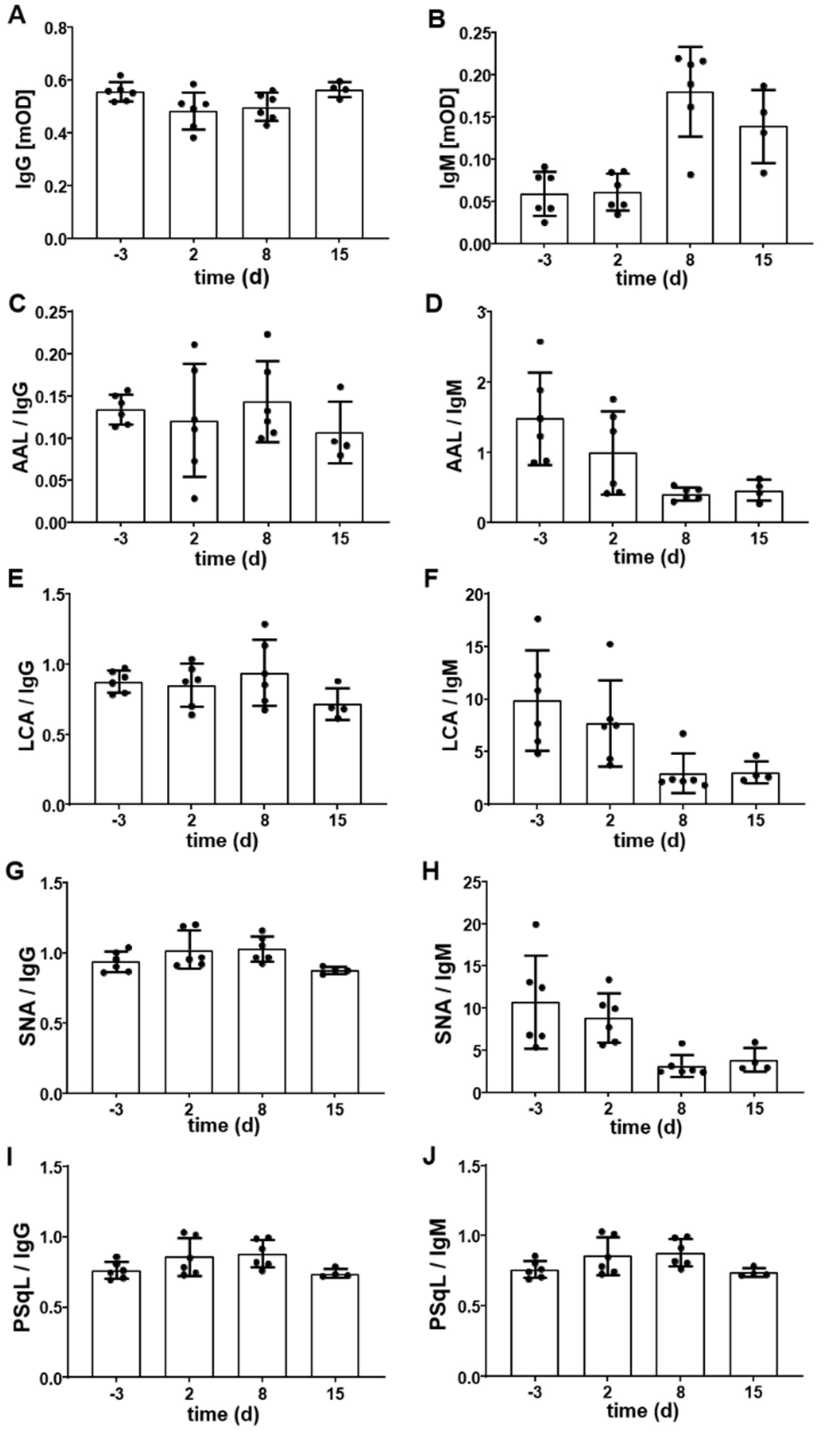

Figure S1. Immunoglobulin-associated glycans change within the course of experimental murine sepsis in Balb/c mice. Time course of the levels of IgG (A) and IgM (B) before and after sepsis induction is displayed. The serum immunoglobulin-associated glycans were detected by the lectins AAL (core α1,6-fucose) (C, D), LCA (fucosylated trimannose) (E, F), SNA (terminal a2,6-sialic acid) (G, H), and PSqL (terminal a 2,6-sialic acid of N-glycans) (I, J). The levels of lectins were normalized to the levels of IgG and IgM. Note, that the glycosylation of both IgG and IgM changed substantially in the course of sepsis. AAL = Aleuria aurantia lectin; LCA = Lens culinaris agglutinin; SNA = Sambucus nigra agglutinin; pSqL = Polyporus squamosus lectin. Kruskall-Wallis test with Dunn's multiple comparisons posttest was used to compare differences among time points (n = 6).

Table S1. Significant changes in the exposure of immunoglobulin-associated glycans within the course of experimental murine sepsis in Balb/c mice. Significant values are displayed in bold. Kruskall-Wallis test with Dunn's multiple comparisons posttest was used to compare differences among time points (n = 6).

time	IgG	IgM	AAL/ IgG	AAL/ IgM	LCA/ IgG	LCA/ IgM	SNA/ IgG	SNA/ IgM	PSqL/IgG	PSqL/IgM
day -3 vs 2	n.s.	n.s.	n.s.	n.s.	n.s.	n.s.	n.s.	n.s.	n.s.	n.s.
day -3 vs 8	n.s.	0.0111	n.s.	0.0130	n.s.	0.0082	n.s.	0.0096	n.s.	0.0130
day -3 vs 15	n.s.	n.s.	n.s.	n.s.	n.s.	n.s.	n.s.	n.s.	n.s.	n.s.
day 2 vs 8	n.s.	0.0266	n.s.	n.s.	n.s.	n.s.	n.s.	0.174	n.s.	0.0266
day 2 vs 15	n.s.	n.s.	n.s.	n.s.	n.s.	n.s.	n.s.	n.s.	n.s.	n.s.
day 8 vs 15	n.s.	n.s.	n.s.	n.s.	n.s.	n.s.	0.0304	n.s.	n.s.	n.s.

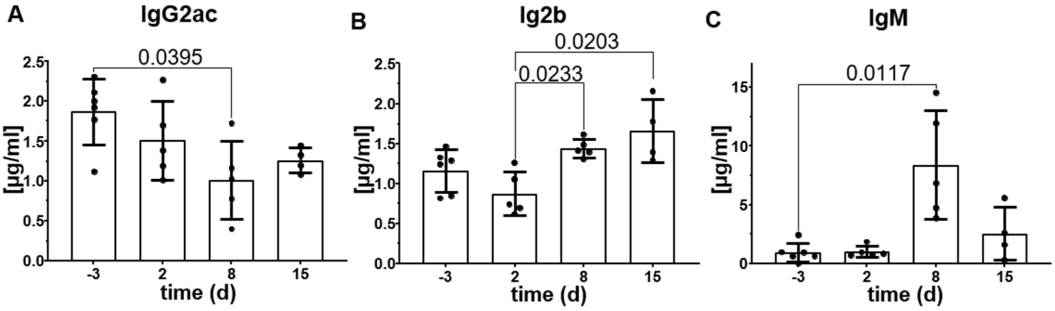

Figure S2. Changes in the serum immunoglobulin levels over the time course of experimental sepsis in Balb/c mice. Serum immunoglobulins (Ig) concentrations were measured by LEGENDplex™ bead assay before and after sepsis induction in Balb/c mice (only significant data are shown). Kruskall-Wallis test with Dunn's multiple comparisons posttest was used to compare differences among time points (n = 6).

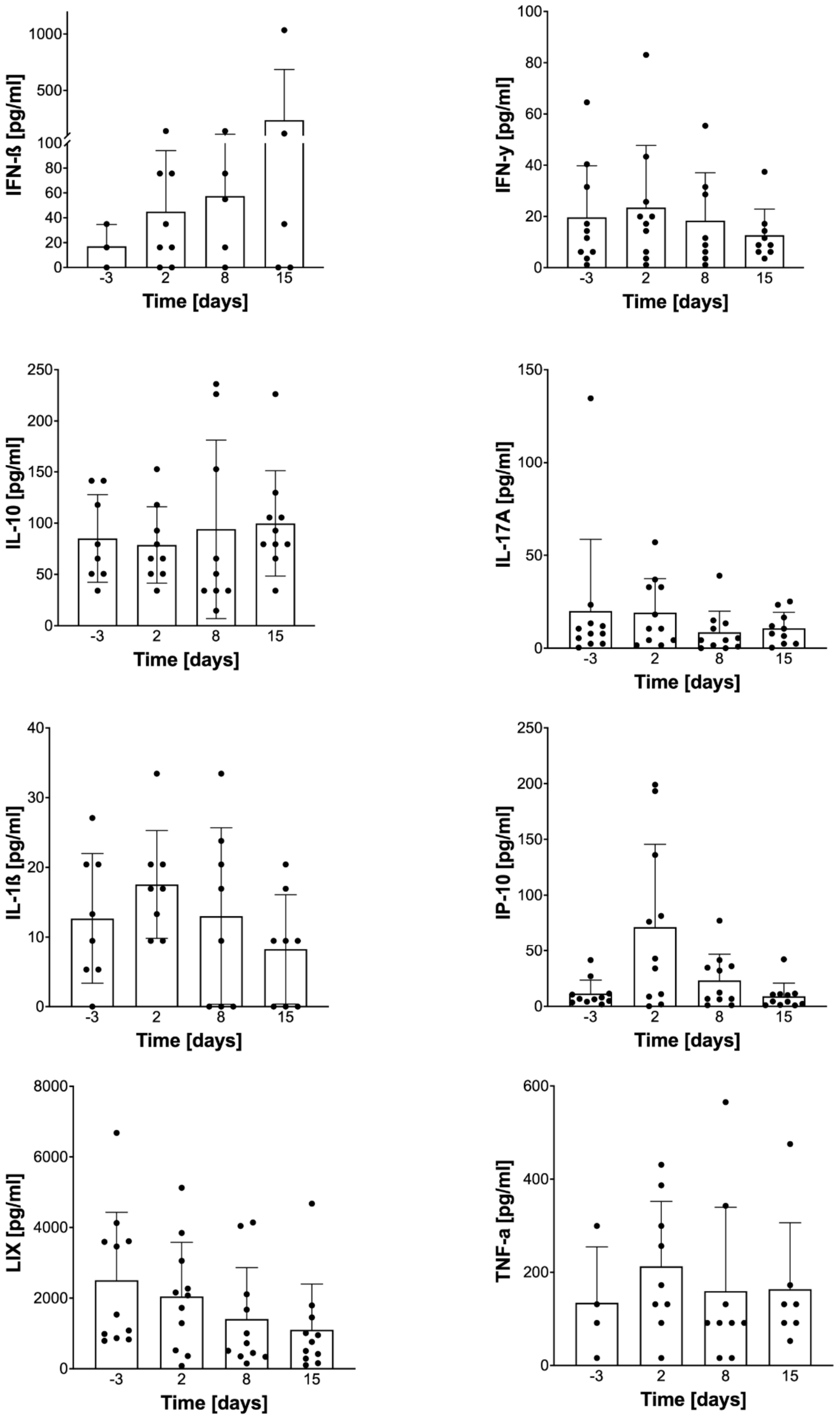

Figure S3. Changes in the serum cytokine/chemokine levels over the time course of experimental sepsis in C57/BL6N mice. Serum cytokine/chemokine concentrations were measured by a custom-made LEGENDplex™ cytokine/chemokine detection assay before and after sepsis induction in C57/BL6N mice. No significant statistical differences have been observed for these cytokines over the time course of experimental sepsis.

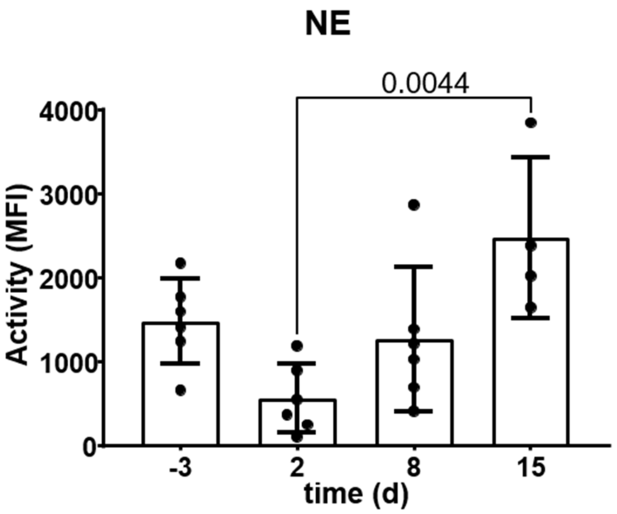

Figure S4. Changes in neutrophils elastase activity the time course of experimental sepsis in Balb/c mice. Changes in the mean fluorescence intensity (MFI) levels of substrate converted by neutrophil elastase (NE) in the sera of Balb/c mice during the course of experimental sepsis over time in days Kruskall-Wallis test with Dunn's multiple comparisons posttest was used to compare differences among time points (n = 6).
